# The difference between subjective symptoms and objective symptom of edema during the menstrual cycle

**DOI:** 10.1038/s41598-026-55554-1

**Published:** 2026-06-01

**Authors:** Yuki Takano, Tatuki Shirai, Yudai Tanaka, Kodai Sakamoto, Hirotake Yokota, Ryo Hirabayashi, Tomonobu Ishigaki, Makoto Komiya, Mutsuaki Edama

**Affiliations:** Institute for Human Movement and Medical Sciences, Niigata University of Health and Welfare, Shimami-cho 1398, Kita-ku, Niigata City, 950-3198 Japan

**Keywords:** Menstrual cycle, Premenstrual syndrome, Edema, Menstrual distress questionnaire, Health care, Medical research, Risk factors

## Abstract

This study aimed to elucidate the differences between subjective and objective symptoms of edema during the menstrual cycle in female university students with regular menstruation. The study included 29 female university students with regular menstrual cycles. The menstrual cycle was categorized into the early follicular, mid-luteal, and late-luteal phases, and measurements were taken during each phase. Subjective symptoms were evaluated using body charts and the Menstrual Distress Questionnaire (MDQ) to assess the presence of edema. Objective symptoms were measured using a body composition analyzer to assess body weight, body water content, and regional water content. In the evaluation of subjective symptoms of edema using the body chart, no significant differences were observed across cycles. However, when body regions were compared, there were significantly fewer subjective symptoms in the upper arm (Rt/Lt) than in the face and trunk across all phases, while the lower leg (Rt/Lt) had significantly more subjective symptoms. The MDQ (water retention item) showed no marked differences across cycles. For objective evaluations of edema, including body weight and total body water, no significant differences were observed across cycles; however, regional water content was significantly higher in the trunk and right arm during the mid-luteal and late-luteal phases than in the early follicular phase. Our findings suggest that the location and timing of subjective edema symptoms may differ from those of objective symptoms.

**Supplementary Information:**

The online version contains supplementary material available at 10.1038/s41598-026-55554-1.

## Introduction

Premenstrual syndrome (PMS) refers to a constellation of emotional, behavioral, and physical symptoms that occur during the late luteal phase of the menstrual cycle and resolve with the onset of menstruation^1^. Such symptoms are generally observed in the late luteal phase, when circulating levels of the ovarian hormone progesterone (P4) are elevated and tend to subside or resolve shortly after menstruation begins—typically within four days. These observations imply that cyclical hormonal variations across the menstrual cycle may influence fluid retention–related symptoms^2^.

The menstrual cycle is controlled by dynamic changes in female steroid hormones^3–5^. These hormonal variations involve the hypothalamic secretion of gonadotropin-releasing hormone (GnRH), the release of follicle-stimulating hormone (FSH) and luteinizing hormone (LH) from the anterior pituitary gland, and the ovarian production of estrogen (E2) and P4^3^. Estrogen promotes the release of vasopressin^6,7^, a key hormone in osmotic regulation, leading to an increase in plasma volume^8^. P4 also contributes to plasma volume expansion^8^. Consequently, both E2 and P4 play crucial roles in the regulation of body fluids, and their cyclical fluctuations throughout the menstrual cycle are likely to influence body fluid regulation and edema-related symptoms.

One method for evaluating menstrual cycle–related symptoms is the Menstrual Distress Questionnaire (MDQ)^9^. The MDQ comprises 46 items rated on a five-point scale based on symptom severity. These items are categorized into eight subscales: pain, water retention, autonomic reactions, negative affect, concentration, behavioral change, arousal, and control, allowing for a comprehensive assessment of menstrual cycle–related symptoms. The water retention subscale, which was the focus of this study, assesses four specific symptoms: weight gain, skin problems, breast pain or tenderness, and breast or abdominal bloating. However, the MDQ is based on subjective self-assessment and does not objectively confirm the presence of edema.

Objective methods to assess edema typically involve quantifying body water content and its distribution. Among these, bioelectrical impedance analysis (BIA) is a widely utilized, non-invasive technique that estimates parameters such as body fat percentage, fat-free mass, and total body water (TBW). Prior research examining fluctuations in body composition throughout the menstrual cycle using BIA has yielded mixed results, with some studies reporting no significant cycle-related changes^10–15^. Conversely, other investigations have documented phenomena such as weight gain during the luteal phase^16,17^, weight reduction in the follicular phase^18^, and elevated TBW in the late luteal phase^19,20^, reflecting inconsistencies in the literature. Recent advancements in BIA technology have improved its accuracy, allowing detailed evaluation of edema, including segmental water content and extracellular water ratio^21–23^. Importantly, most previous studies have evaluated either subjective symptoms or objective body composition changes separately. To our knowledge, few studies have directly compared subjective perceptions of edema with objective segmental body water measurements across clearly defined menstrual cycle phases within the same cohort. This direct comparison represents the primary novelty and contribution of the present study.

This study focuses on general menstrual cycle–related changes in edema and examines the discrepancy between subjective perceptions of edema and objectively measured body water across clearly defined menstrual cycle phases in healthy female university students with regular menstrual cycles.

## Results

Their characteristics were as follows: age 20.2 ± 0.7 years, height 162.8 ± 5.9 cm, and weight 55.5 ± 7.5 kg, body mass index 21.9 ± 5.0 (kg/m^2^).

### E2 and P4 concentrations across menstrual cycles

The results for E2 and P4 concentrations during each cycle phase are presented in Table 1. For E2 concentrations, no significant main effects were observed in the repeated-measures analysis of variance. However, for P4 concentrations, a significant main effect of the menstrual cycle on hormone levels was found (F (1.574, 44.067) = 4.752, p = 0.020, partial η^2^ = 0.145, observed power = 0.695). Post-hoc Tukey’s test revealed a significant difference between the early follicular and mid-luteal phases (p = 0.009, mean difference = 140.68, 95% CI [29.91–251.45]).

**Table 1:** Changes in estradiol and progesterone concentration during the menstrual cycle

	Early follicular phase	Middle luteal phase	Late luteal phase	Total	*F* value	*P* value	Partial η^2^	Power
Estradiol [pg/mL]	1.3 ± 0.4	1.4 ± 0.6	1.3 ± 0.7	1.3 ± 0.5	(1.616, 45.261) 0.864	0.407	0.030	0.175
Progesterone [pg/mL]	217.7 ± 132.3	358.4 ± 302.3^a^	303.7 ± 250.1	293.3 ± 243.3	(1.574, 44.067) 4.752	0.020	0.145	0.695

N = 29

Values are presented as means ± SD.

^*a*^Statistically significant difference compared with the early follicular phase (*p* = 0.009, mean difference = 140.68=140.68, 95%CI[29.91–251.45]) .

#### Evaluation of subjective symptoms of edema: Body chart and MDQ results

The results of the body chart and MDQ (fluid retention items) are presented in Tables 2 and 3, respectively. In the body chart analysis, Pearson’s chi-square test revealed no significant differences across menstrual cycle phases. However, between the body regions, edema was significantly less frequent in the upper arms (right/left) than in the face and trunk (p < 0.001) and more frequent in the lower legs (right and left) (*p* < 0.001) across all phases. For the MDQ (fluid retention items), no significant main effects were observed across menstrual cycle phases.

**Table 2:** Changes in subjective assessment of edema (body chart) during the menstrual cycle

	Early follicular phase	Middle Luteal phase	Late Luteal phase
Face and Trunk	7 (24.1)	6 (20.7)	5 (17.2)
Upper arm (Rt)	2 (6.9)^a^	1 (3.4)^e^	2 (6.9)^i^
Upper arm (Lt)	3 (10.3)^b^	2 (6.9)^f^	1 (3.4)^j^
Lower leg (Rt)	18 (62.1)^c^	12 (41.4)^g^	16 (55.2)^k^
Lower leg (Lt)	18 (62.1)^d^	12 (41.4)^h^	17 (58.6)^l^

N=29

Number(%)

^*a*^Statistically significant difference compared with face and trunk in the early follicular phase (*P* < .001, adjusted residuals [ −3.4, 3.4]).

^*b*^Statistically significant difference compared with face and trunk in the early follicular phase (*P* < .001, adjusted residuals [ −2.9, 2.9]).

^*c*^Statistically significant difference compared with face and trunk in the early follicular phase (*P* < .001, adjusted residuals [3.7,  −3.7]).

^*d*^Statistically significant difference compared with face and trunk in the early follicular phase (*P* < .001, adjusted residuals [3.7,  −3.7]).

^*e*^Statistically significant difference compared with face and trunk in the middle luteal phase (*P* < .001, adjusted residuals [ −2.7, 2.7]).

^*f*^Statistically significant difference compared with face and trunk in the middle luteal phase (*P* < .001, adjusted residuals [ −2.7, 2.7]).

^*g*^Statistically significant difference compared with face and trunk in the middle luteal phase (*P* < .001, adjusted residuals [2.8,  −2.8]).

^*h*^Statistically significant difference compared with face and trunk in the middle luteal phase (*P* < .001, adjusted residuals [2.8,  −2.8]).

^*i*^Statistically significant difference compared with face and trunk in the late luteal phase (*P* < .001, adjusted residuals [ −2.9, 2.9]).

^*j*^Statistically significant difference compared with face and trunk in the late luteal phase (*P* < .001, adjusted residuals [ −3.3, 3.3]).

^*k*^Statistically significant difference compared with face and trunk in the late luteal phase (*P* < .001, adjusted residuals [3.6,  −3.6]).

^*l*^Statistically significant difference compared with face and trunk in the late luteal phase (*P* < .001, adjusted residuals [4.1,  −4.1]).

**Table 3:** Changes in subjective assessment (Water retention of MDQ) during the menstrual cycle

	Early follicular phase	Middle Luteal phase	Late Luteal phase
Water retention	3.6 ± 3.1	2.1 ± 2.5	2.8 ± 3.1

Values are presented as means ± SD.

SD: Standard Deviation

#### Objective evaluation of edema: InBody measurements results

The results for weight (Kg), TBW (L), segmental water content (L), ECW (L), and ECW/TBW ratio are presented in Table 4. Repeated measures analysis of variance revealed no significant differences across menstrual cycle phases for weight, TBW, ECW, and ECW/TBW ratio. However, significant main effects were observed for segmental water content in the trunk (F (2, 59) = 4.919, *p* = 0.011, partial η^2^ = 0.149, observed power = 0.786) and right upper arm (F (2, 56) = 5.356, *p* = 0.007, partial η^2^ = 0.161, observed power = 0.821). Post-hoc Tukey’s test revealed that the trunk water content, values were significantly higher in the mid luteal phase than in the early follicular phase (*p* = 0.034, mean difference = 0.1241, 95% CI [0.0075–0.2408]) and in the late luteal phase than in the early follicular phase (*p* = 0.017, mean difference = 0.1379, 95% CI [0.0213–0.2546]) . In addition, for the right upper arm water content was significantly higher in the mid-luteal phase compared to the early follicular phase (p = 0.023, mean difference = 0.0262, 95% CI [0.003–0.049]) and in the late luteal phase compared to the early follicular phase (p = 0.013, mean difference = 0.0283, 95% CI [0.005–0.051]).

**Table 4:** Changes in objective assessment (in the body) during the menstrual cycle

	Early follicular phase	Middle luteal phase	Late luteal phase	F value	*P* value	Partial η^2^	Power
Body Weight(kg)	55.55 ± 7.51	55.40 ± 7.59	55.55 ± 7.66	(2, 56) 0.566	0.571	0.020	0.139
Total Body Water(L)	30.36 ± 4.18	30.53 ± 4.49	30.64 ± 4.27	(2, 056) 2.638	0.080	0.086	0.504
*Segmental Body Water(L)*							
Trunk	13.93 ± 1.83	14.05 ± 1.88^a^	14.07 ± 1.80^b^	(2, 59) 4.919	0.011	0.149	0.786
Right Arm	1.50 ± 0.30	1.53 ± 0.31^c^	1.53 ± 0.30^d^	(2, 56) 5.356	0.007	0.161	0.821
Left Arm	1.47 ± 0.29	1.51 ± 0.31	1.49 ± 0.29	(1.285, 35.970) 2.486	0.116	0.082	0.377
Right Leg	5.05 ± 0.76	5.08 ± 0.81	5.10 ± 0.79	(2, 6) 1.960	0.150	0.065	0.389
Left Leg	5.03 ± 0.76	5.07 ± 0.81	5.07 ± 0.78	(2, 56) 1.753	0.183	0.059	0.352
Extracellular Water(L) ECW/TBW	11.45 ± 1.46 0.38 ± 0.01	11.49 ± 1.61 0.37 ± 0.02	11.57 ± 1.51 0.38 ± 0.01	(2, 56) 2.030 (1.037, 29.045) 1.539	0.141 0.226	0.068 0.052	0.401 0.228

Values are presented as means ± SD.

ECW/TBW: Extracellular Water to Total Body Water Ratio

SD: Standard Deviation

^a^Statistically significant difference compared with the early follicular phase (*p* = 0.034, mean difference=0.1241, 95%CI[0.0075–0.2408]) .

^b^Statistically significant difference compared with the early follicular phase (*p* = 0.017, mean difference=0.1379, 95%CI[0.0213–0.2546]) .

^c^Statistically significant difference compared with the early follicular phase (*p* = 0.023, mean difference=0.0262, 95%CI[0.003–0.049]) .

^d^Statistically significant difference compared with the early follicular phase (*p* = 0.013, mean difference=0.0283, 95%CI[0.005–0.051]) .

## Discussion

Our findings revealed that subjective measurements did not show cyclical changes, with edema more frequently observed in the lower limbs. In contrast, objective measurements revealed significantly higher segmental body water content in the trunk and right upper arm during the luteal phases (mid-luteal and late luteal phases) compared to the early follicular phase. These findings suggest a disconnect between subjective edema symptoms and physiological changes. Participants in this study were not screened or diagnosed for premenstrual syndrome (PMS). PMS is discussed here solely to provide contextual background regarding menstrual cycle–related symptoms.

Subjective symptoms of edema during the menstrual cycle have been frequently reported in studies on PMS and Premenstrual Dysphoric Disorder, often including complaints of edema and abdominal bloating, which typically appear in the late luteal phase of the menstrual cycle and subside with the onset of menstruation^1,9,24^. However, the results of the MDQ in this study showed no significant changes in fluid retention items across the menstrual cycle. Moreover, the body chart revealed no significant differences between cycles; however, edema was significantly more frequent in the lower legs (Rt/Lt) than in the face and trunk throughout the entire cycle, contradicting the results of previous studies. This discrepancy may reflect not only PMS symptoms but also primary dysmenorrhea-related symptoms such as abdominal bloating and fatigue^25,26^.

Further, objective evaluations of edema, such as body water content and limb circumference, do not align with subjective symptoms, and while changes in body weight and body water content have been observed, no direct correlation with the sensation of swelling has been reported^16,27^. Similarly, this study found increased body water content in the trunk and right upper arm during the mid-luteal and late-luteal phases. Interestingly, this effect was observed only in the right upper arm and not in the left. One possible explanation may be limbing dominance, as most participants were likely right-handed, potentially leading to subtle differences in muscle mass or fluid distribution. Alternatively, this asymmetry may reflect measurement variability inherent to segmental BIA or minor physiological differences that were not captured in this study. Therefore, the observed unilateral change should be interpreted with caution. However, subjective symptoms remained consistent across cycles, with edema most frequently observed in the lower limbs, indicating a discrepancy between objective and subjective evaluations. This discrepancy may stem from the regulatory effects of estrogen and progesterone on water and electrolyte balance. Stachenfeld et al.^6–8^ have reported that these hormones affect antidiuretic hormone (AVP) and sodium excretion, leading to changes in plasma and extracellular fluid volumes. In this study, the salivary P4 concentration was significantly higher in the mid-luteal phase compared to the follicular phase. Therefore, body water changes probably occur, but the sensation of edema does not align with them. In addition, Cumberledge et al.^10^ and Hicks et al.^11^ have noted that body water measurements using BIA have limitations in detecting minor changes in body water caused by hormonal fluctuations, suggesting that the limitations may contribute to the discrepancy between objective and subjective symptoms.

Furthermore, the intensity of subjective symptoms is believed to be influenced by psychological factors, and fluctuations in hormone levels are reportedly strongly associated with emotional changes and sensitivity to bodily sensations^28,29^. In this study, only salivary levels of E2 and P4 hormones were analyzed; however, hormonal fluctuations throughout the menstrual cycle may affect the perception, potentially leading individuals to misinterpret other symptoms or factors as edema.

Our findings are clinically valuable. For example, when young women report edema, it is important not only to evaluate the presence of fluid retention but also to consider the menstrual cycle and psychological state comprehensively. In addition, in exercise instruction and nutritional management, understanding subjective reports of bodily changes and responding appropriately could serve as evidence to guide interventions.

This study has several limitations. First, the participants were limited to healthy female university students with regular menstrual cycles and no hormonal contraceptive use, and they were not formally screened for PMS or dysmenorrhea. Therefore, the findings are most applicable to women with similar characteristics and should not be directly generalized to clinical populations or other age groups. Second, although the sample size was modest (n = 29), a priori power estimation indicated that it was sufficient to detect moderate within-subject effects. However, smaller effect sizes, particularly in subjective symptom measures—may not have been detected. Third, menstrual cycle phases were determined based on basal body temperature and self-report, and hormone levels were assessed using saliva samples. In addition, the early follicular phase overlapped with menstruation, during which dysmenorrhea-related symptoms may have influenced measurements. Furthermore, bioelectrical impedance analysis (BIA) has inherent limitations in detecting subtle or localized changes in body fluid distribution. Despite these limitations, the within-subject repeated-measures design strengthened internal validity and

## Materials and methods

### Participants

**Fig. 1:**
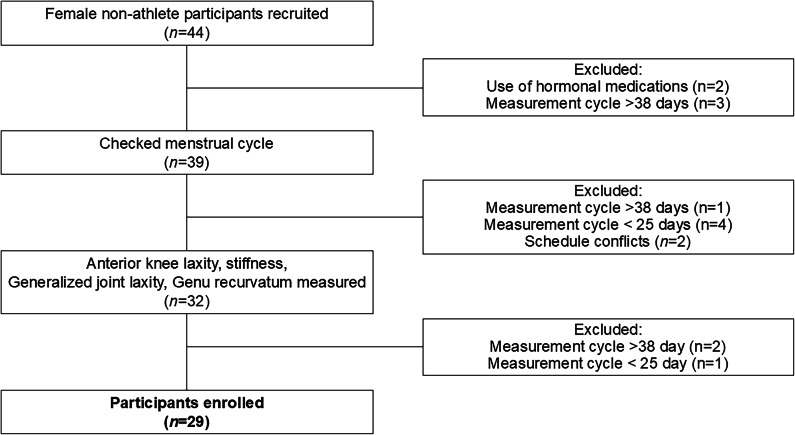
Flowchart for the selection of the female participants.

Participants were recruited using convenience sampling from female university students at our institution between December 2022 and June 2023. Study information was provided both verbally and in written form during lectures. Students who expressed interest were screened for eligibility according to predefined inclusion and exclusion criteria. A questionnaire survey was administered to 44 female university students, of whom 29 met the inclusion criteria: a menstrual cycle length of 25–38 days^30^, and no use of oral contraceptives or hormonal agents within the past 6 months^31^. Exclusion criteria included irregular menstrual cycles, use of medications affecting hormonal status or fluid balance, and any known gynecological or endocrine disorders. All participants had regular menstruation and provided informed consent to participate in the study (Fig. 1). All study protocols were performed according to the Declaration of Helsinki, after receiving approval from the ethics committee at our institution (approval number: 18831-220526). Written informed consent was obtained from all participants prior to enrollment following both verbal and written explanations of the study purpose, procedures, risks, and confidentiality.

#### Recording of the menstrual cycle

Participants were instructed to measure their basal body temperature (BBT) every morning upon waking, beginning 1–2 months prior to the experiment. BBT was measured

orally (sublingually) immediately after waking using a digital basal thermometer (Citizen Electronic Thermometer CTEB503L, Citizen Systems Co., Ltd.). To estimate the day of ovulation, participants were provided with ovulation test kits (Doctor’s Choice One Step Ovulation Test Clear, Beauty & Health Research Inc.) and were asked to use them daily starting from the day after menstruation stopped and until a positive result was obtained. Participants recorded their daily BBT, menstruation period, and ovulation test results using the ONE TAP SPORTS conditioning management system (Euphoria Co., Ltd.)^32,33^.

#### Timing of measurements

Measurements were conducted once during each of the phases (the early follicular, mid-luteal, and late-luteal phases), totaling three measurements per participant. The analysis focused on estradiol (E2) and progesterone (P4) levels. The early follicular phase, characterized by low levels of both E2 and P4; the mid-luteal phase, with high levels of both hormones; and the late-luteal phase, with low E2 and high P4 levels, were selected as the periods of interest^3^. These three phases were chosen to represent distinct hormonal environments across the menstrual cycle, enabling comparison between low-hormone and high-hormone states and examination of potential hormone-related changes in body fluid regulation. The early follicular phase was defined as 2–4 days following the onset of menstruation^34^, the mid-luteal phase was defined as 2–4 days following a positive ovulation test^5^, and the late-luteal phase as 12 days following a positive ovulation test until the day before the next menstruation^29^. To account for diurnal variation, all measurements were conducted between 8:00 AM and 12:00 PM^35^.

### Measurement methods

#### Concentrations of estradiol and progesterone

Salivary concentrations of estradiol (E2) and progesterone (P4) were measured. To minimize potential influences on hormone levels, participants were instructed to strictly adhere to six precautions as described in previous studies^33^: (1) no alcohol consumption within 12 h before sampling; (2) no food intake within 60 min; (3) no tooth brushing within 45 min; (4) no dairy products within 20 min; (5) no intake of sugary, acidic, or caffeinated beverages before sampling; and (6) no saliva collection within 48 h after dental treatment. In addition, participants were instructed to rinse their mouths before the experiment to remove any food residue. To prevent a decrease in hormone levels, saliva was collected at least 10 min after rinsing. Participants held saliva in their mouths for 1 min and then transferred it into a collection vial (Cryovial, SAL) using a dedicated straw (Saliva Collection Aid, SAL). Saliva samples were immediately stored in a freezer at –80°C. After all samples were collected, hormone analysis was outsourced to Funakoshi Co., Ltd.

The concentrations of estradiol (E2) were analyzed using a High Sensitivity Salivary 17β-Estradiol Enzyme Immunoassay Kit (Salimetrics LLC, State College, PA, USA), and progesterone (P4) concentrations were analyzed using a Salivary Progesterone Enzyme Immunoassay Kit (Salimetrics LLC, State College, PA, USA). The samples were thawed at room temperature, mixed using a vortex mixer, centrifuged at 1500 × g for 15 min, and analyzed using enzyme-linked immunosorbent assay. All samples were analyzed without dilution (i.e., 1× concentration)^32,33^.

#### Subjective symptoms of edema

Subjective symptoms of edema were assessed using two methods. First, a body chart was used to identify the perceived locations of edema. Participants were asked to check the areas where they experienced symptoms of edema, including the face, trunk, right and left upper arms, forearms, hands (including fingers), right and left thighs, lower legs, and feet (including toes). The checked regions were then grouped and summed as follows: trunk (face and trunk), right and left upper limbs (upper arm, forearm, and hand), and right and left lower limbs (thigh, lower leg, and foot). Second, the MDQ^9^ was used to assess four items related to fluid retention associated with PMS: weight gain, skin problems, breast pain/tenderness, and swelling in the breasts or abdomen. Each item was rated on a 5-point scale ranging from 0 to 4, where 0 = not at all, 1 = mild, 2 = moderate, 3 = severe, and 4 = very severe. Higher scores indicated more intense subjective symptoms.

The Menstrual Distress Questionnaire (MDQ) is a widely used and validated instrument for assessing menstrual cycle–related symptoms. Previous studies have demonstrated its acceptable internal consistency and construct validity. In the present study, only the water retention subscale was analyzed.

The body chart used to assess perceived edema locations was developed for this study based on anatomical segmentation and was used as a descriptive tool to identify perceived edema distribution.

#### Objective symptom of edema

Objective symptoms of edema were assessed using the InBody970 (InBody Co., Ltd.). Participants were instructed to follow three pre-measurement guidelines: (1) refrain from eating for at least 2 h before measurement, (2) urinate prior to the assessment, and (3) remove all metal accessories. All measurements were conducted between 8:00 AM and 12:00 PM to minimize diurnal variation. Breakfast intake was not standardized across participants due to practical constraints; however, participants were required to maintain at least a 2-hour fasting period before testing. Short-term factors such as recent fluid intake or bathing were not fully controlled. The parameters measured by the InBody970 included body weight, TBW, segmental water content (trunk, right and left upper limbs, and right and left lower limbs), extracellular water (ECW), and the ratio of ECW to TBW (ECW/TBW). These measurements were conducted during each menstrual cycle phase.

### Statistical analysis

Continuous variables are expressed as mean ± standard deviation (SD). To examine the differences in hormone concentrations, MDQ scores, and InBody measurements (body weight, TBW, segmental water content, ECW, and ECW to TBW ratio) across menstrual cycle phases, a one-way repeated measures analysis of variance (ANOVA) was conducted. When a significant main effect was found, Tukey’s post-hoc test was performed. For subjective edema assessed using the body chart, chi-square tests were used to compare differences across body regions and menstrual phases. The significance level was set at 5%.

Sample size estimation was performed a priori using G*Power (version 3.1; test family: F tests; statistical test: ANOVA: repeated measures, within factors). Assuming three measurements (early follicular, mid-luteal, and late-luteal phases), an effect size of Cohen’s f = 0.25, α = 0.05, power (1−β) = 0.80, correlation among repeated measures = 0.50, and ε = 1.0, the required total sample size was 28 participants. The assumed effect size was based on expected within-subject physiological changes across menstrual phases and was consistent with the observed effect sizes in the present study. To account for potential dropouts or missing data, we aimed to recruit at least 28 participants, and 29 participants were included in the final analysis.

## Conclusion

This study examined the differences between subjective symptoms of edema and changes in body water content during the menstrual cycle in healthy female university students with normal menstrual cycles. Our findings indicate that subjective and objective symptoms of edema differ in timing and location. Future studies should focus more on diverse age groups, women with different lifestyles, objective hormone level measurements, and precise methods for assessing body fluid distribution.

## Supplementary Information

Below is the link to the electronic supplementary material.

Supplementary Material 1

Supplementary Material 2

## Data Availability

The datasets generated and/or analyzed during the current study are not publicly available because of limitations of ethical approval involving patient data and anonymity but are available from the corresponding author upon reasonable request.
