# Supplementary material for: The difference between subjective symptoms and objective symptom of edema during the menstrual cycle
Source: Sci Rep. 2026 Jun 1;16:25009. doi: 10.1038/s41598-026-55554-1 (PMC13462954; doi:10.1038/s41598-026-55554-1)
Supplement: Supplementary file 1 [file 41598_2026_55554_MOESM1_ESM.docx]

# Supplementary File 1

## Water Retention Items from the Menstrual Distress Questionnaire (MDQ)

Water retention items assessed in this study were derived from the Menstrual Distress Questionnaire (MDQ; Moos, 1968). The following four items were used in the present study:

1. 1. Weight gain
2. 2. Skin problems
3. 3. Breast pain or tenderness
4. 4. Breast or abdominal bloating

Each item was rated on a 5-point Likert scale:

- 0 = Not at all
- 1 = Mild
- 2 = Moderate
- 3 = Severe
- 4 = Very severe

Note: The full MDQ questionnaire is copyrighted by Mind Garden, Inc. Only the items analyzed in this study are presented here. The complete instrument is available from www.mindgarden.com.
